# Cytoscape Web: bringing network biology to the browser

**DOI:** 10.1093/nar/gkaf365

**Published:** 2025-05-01

**Authors:** Keiichiro Ono, Dylan Fong, Chengzhan Gao, Christopher Churas, Rudolf Pillich, Joanna Lenkiewicz, Dexter Pratt, Alexander R Pico, Kristina Hanspers, Yihang Xin, John Morris, Mike Kucera, Max Franz, Christian Lopes, Gary Bader, Trey Ideker, Jing Chen

**Affiliations:** Department of Medicine, University of California, San Diego, CA 92093, United States; Department of Medicine, University of California, San Diego, CA 92093, United States; Department of Medicine, University of California, San Diego, CA 92093, United States; Department of Medicine, University of California, San Diego, CA 92093, United States; Department of Medicine, University of California, San Diego, CA 92093, United States; Department of Medicine, University of California, San Diego, CA 92093, United States; Department of Medicine, University of California, San Diego, CA 92093, United States; Data Science and Biotechnology, Gladstone Institutes, San Francisco, CA 94158, United States; Data Science and Biotechnology, Gladstone Institutes, San Francisco, CA 94158, United States; Data Science and Biotechnology, Gladstone Institutes, San Francisco, CA 94158, United States; Resource for Biocomputing, Visualization and Informatics, University of California, San Francisco, CA 94118, United States; The Donnelly Centre, University of Toronto, Ontario M5S 3E1, Canada; The Donnelly Centre, University of Toronto, Ontario M5S 3E1, Canada; The Donnelly Centre, University of Toronto, Ontario M5S 3E1, Canada; The Donnelly Centre, University of Toronto, Ontario M5S 3E1, Canada; Department of Medicine, University of California, San Diego, CA 92093, United States; Department of Medicine, University of California, San Diego, CA 92093, United States

## Abstract

Since its introduction in 2003, Cytoscape has been a *de facto* standard for visualizing and analyzing biological networks. We now introduce Cytoscape Web (https://web.cytoscape.org), an online implementation that captures the interface and key visualization functionality of the desktop while providing integration with web tools and databases. Cytoscape Web enhances accessibility, simplifying collaboration through online data sharing. It integrates with Cytoscape desktop via the CX2 network exchange format and with the Network Data Exchange for storing and sharing networks. The platform supports extensibility through an App framework for UI components and Service Apps for algorithm integration, fostering community-driven development of new analysis tools. Overall, Cytoscape Web enhances network biology by providing a versatile, accessible, and collaborative online platform that adapts to evolving computational challenges, laying a foundation for future incorporation of advanced network analysis capabilities by the community.

## Graphical Abstract

**Graphical Abstract:**
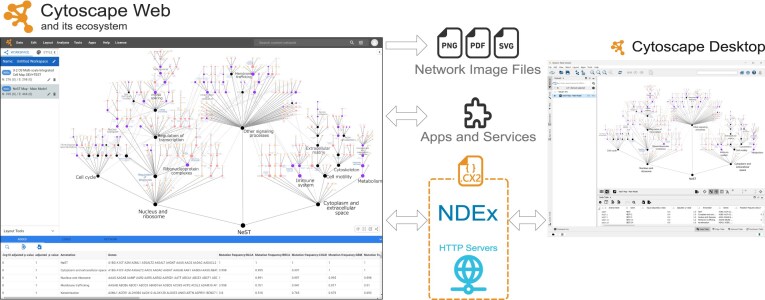

## Introduction

As biomedical data grow larger and more complex, researchers increasingly rely on network-based analysis tools to understand the many interactions among genes, proteins, disease phenotypes, drugs, and other interconnected biological or clinical entities. Network analysis has been essential in structural biology for mapping protein complexes and cell–cell interactions, in functional biology for mapping regulatory networks and complex biomarkers, and in the study of disease via drug and patient interactions. Since its introduction in 2003, a primary hub for biomedical network analysis and visualization has been Cytoscape [1] (https://cytoscape.org), with over 300 000 downloads annually. Cytoscape provides tools for visualizing, exploring, and analyzing complex biological networks, including support for diverse molecular and omics data types, as well as access to a variety of interaction databases. Users can tailor the appearance and layout of networks by mapping quantitative and categorical attributes to node and edge properties such as size, color, shape, and connectivity.

A primary driver of Cytoscape’s widespread adoption is its user-friendly interface, data-driven visual styles, and flexible architecture supported by a community-driven and extensive ecosystem of Apps. Cytoscape provides programmatic access through representational state transfer application programming interfaces (REST APIs), making it suitable for advanced users and developers.

Complementing the Cytoscape platform are other network analysis tools such as NetworkX [2] and igraph [3], which are popular choices among bioinformaticians and data scientists working in scripting languages such as Python. Web-based platforms including Gephi-lite (https://gephi.org/gephi-lite/) and GraphSpace [4] have recently demonstrated the potential of browser-based network tools. Specialized editors like Newt [5] and NDExEdit (https://frankkramer-lab.github.io/NDExEdit) [6] showcase strengths of these tools in areas such as pathway editing, network manipulation, and public sharing. These advances illustrate the promise of fully featured, web-based solutions for network biology, with each tool targeting specific use cases or data formats. In parallel, the rapid advancement of web technologies and availability of open-source libraries allow modern browsers to provide robust platforms for data visualization and analysis, tasks that in the past were only possible via desktop applications. Researchers increasingly need platform-independent tools that integrate with big data and cloud-based resources. These trends call for an expanded Cytoscape ecosystem that enables users to access network visualization and analysis functions on the web as easily as they can on the desktop.

To address these needs, we developed Cytoscape Web (https://web.cytoscape.org), a new web-based tool that brings Cytoscape’s core functionalities into the browser. Cytoscape Web implements these functions using web-based software and service components we previously developed, such as Cytoscape.js [7] for visualization and the Network Data Exchange (NDEx) [8–10] for cloud network storage and management. We describe the architectural details of Cytoscape Web, including principles of system design, architectural overview, data models and core components, Cytoscape eXchange (CX2) format, and system extensibility, as well as elements of the user interface (UI). We show how Cytoscape Web can be used with three examples: two oriented to biological network analysis users and one to bioinformatics service developers. Finally, we use the “Discussion” section to highlight implications of some of the core features, address current limitations, and outline future directions for extending Cytoscape Web. We intend Cytoscape Web to be part of an extensive ecosystem of interoperable web, scripting, programming, and desktop tools that enable users to access network visualization and analysis from any computing environment.

## Materials and methods

### Cytoscape Web system design principles

The system architecture of Cytoscape Web is founded on three core design principles: (i) delivering advanced network analysis and visualization functionality in web browsers, (ii) ensuring integration with the established Cytoscape ecosystem, and (iii) maintaining system stability and extensibility through both Apps and external services.

Building upon Cytoscape Desktop’s network analysis and visualization capabilities developed over two decades, Cytoscape Web implements a single-page application architecture [11] that facilitates data visualization and sharing in modern research environments. The implementation uses React (https://react.dev/), an industry-standard framework for component-based web applications, enabling researchers to leverage established desktop tools alongside modern web technologies. By emphasizing client-side processing, the implementation achieves high level of performance and responsiveness within the browser environment while reducing server-side load to improve system scalability.

Integration with the Cytoscape ecosystem remains a fundamental design priority, centered on the data-driven network visualization approach through data-driven visual styles—a key feature that contributed to Cytoscape Desktop’s popularity. The system implements this core functionality in the web environment while maintaining conceptual alignment with Desktop through standardized data formats and familiar UI patterns, ensuring accessibility for both experienced and new users.

Cytoscape Web continues Cytoscape Desktop’s extensibility model, centered around its App framework, fostering an ecosystem of third-party applications [12–15]. It implements system expandability through two distinct approaches: Apps and Service Apps. Apps are plugin-based modular applications with custom UI components using TypeScript or JavaScript, providing detailed control over user experience design and functionality. The second approach introduces the Service Apps framework, which facilitates integration with computationally intensive external services such as graph layout or clustering. This architecture particularly supports the polyglot environment common in bioinformatics [16, 17], enabling incorporation of tools developed in widely used programming languages. In a Service App, developers implement tools as web services that both perform analyses and specify their UIs to Cytoscape Web as configurations of menus, dialogs, and other standard controls. All that is required is a backend service, eliminating the need for web application development expertise. Algorithms written in commonly used languages such as Python or R can be readily deployed and integrated. This dual approach to expandability ensures Cytoscape Web can accommodate diverse development requirements while maintaining accessibility for both developers and researchers.

### Architectural overview

Cytoscape Web’s React-based architecture uses client-side processing capabilities while reducing backend dependencies. The backend serves exclusively as a service layer for data persistence and authentication. It uses the CX2 format, which is a standardized JavaScript Object Notation (JSON) structure for exchanging network, table, and style data. This architecture allows core tasks—such as network visualization and layout—to be executed directly in the browser, improving responsiveness and reducing reliance on server-side computation.

The client-side architecture handles all core functionalities, leveraging both central processing unit (CPU) and graphics processing unit (GPU) processing capabilities of modern browsers. Network and table data manipulation, visual styling, and user interactions are processed through standard CPU operations, while computationally intensive tasks such as graph layout algorithms or network rendering use GPU acceleration, without any user-side configurations. The backend’s responsibilities are limited to functions that cannot be performed on the client side: serving application code bundles, managing authentication, and storing networks and user’s workspace snapshots such that they are persistent between work environments (e.g. home or office computers). This thin-backend architecture improves scalability by offloading computation to client machines and minimizing server–client data transmission. Currently implemented through NDEx, this approach enables deployment of Cytoscape Web in resource-constrained environments while supporting multiple concurrent users through client-side processing.

### Data models and core components

**Figure 1.**
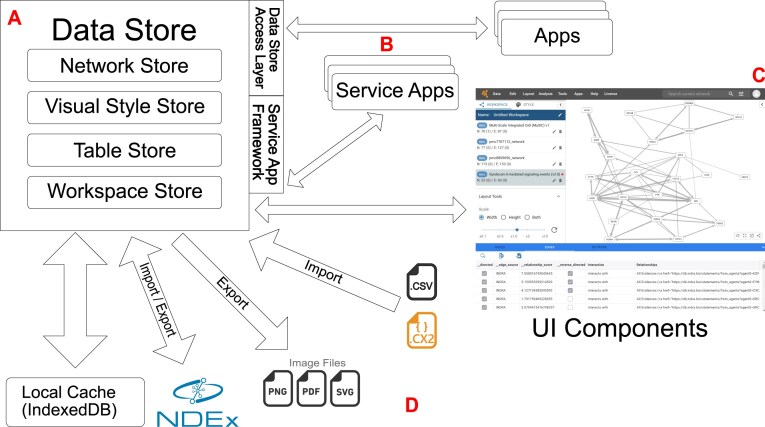
Core components and data flow of Cytoscape Web. (**A**) The central data store manages networks, associated tables, and visual styles in memory, with continuous synchronization to the browser’s IndexedDB to prevent data loss. (**B**) The extensibility layer enables system expansion through two mechanisms: Apps utilizing Module Federation (https://webpack.js.org/concepts/module-federation/) to access the data store and Service Apps that process external service interactions through JSON-defined interfaces. (**C**) The UI layer comprises React components for network management, visualization, and data table manipulation. (**D**) Data persistence and exchange are implemented through NDEx integration for network and workspace storage, with support for direct import of local CSV and CX2 files, and export capabilities for various image formats.

The client-side architecture of Cytoscape Web implements four primary components, mirroring the traditional Cytoscape Desktop system (Fig. 1): central data store, visualization and UI, and data import and export module (Fig. 1A) described below, and the external App framework (“System extensibility” section, Fig. 1B).

The foundation consists of a centralized data store implemented using Zustand (https://github.com/pmndrs/zustand), which manages four core data models: networks, tables, visual styles, and workspace (Fig. 1A). The network model maintains graph topology independent of its implementation, currently using Cytoscape.js for in-memory graph representation. Associated with network elements are data tables that serve as general-purpose storage for node and edge properties, enabling flexible attribute management across the system. Visual styles define both default values and mapping functions that transform tabular data into visual properties such as color, size, and shape. This standardized approach to visual mapping ensures consistency between desktop and web environments. The workspace model encapsulates the application state, including networks, tables, and visual styles, and enables session restoration after page reloads, supporting persistent user workflows. This centralized data architecture provides consistent access across all system components while maintaining efficient state management. The separation of core data models enables flexible implementation of visualization and analysis features while preserving data integrity throughout the system. The visualization and UI components are implemented with React-based modules that manage user interaction and data display (Fig. 1C). Core components include the network manager for workspace organization, style editor for visual mapping configuration, network renderers for graph visualization, and table browser for attribute management. These components interact directly with the central data store to maintain consistent data representation across the interface. The system’s visualization architecture accommodates diverse network structures through pluggable rendering engines and layout algorithms.

The data import/export module maintains data compatibility with Cytoscape Desktop via CX2 while extending web-specific capabilities (Fig. 1D). The module facilitates bidirectional data exchange with NDEx and web servers through the CX2 format, supports network and attribute import from tabular data, and enables export of images in various formats including vector-based PDF. The system also provides workspace persistence through NDEx integration. To maintain data locally, the architecture implements a caching layer using IndexedDB, which preserves core data models across browser sessions. This design enables workspace recovery following browser reloads or application closure while supporting basic offline capabilities with minimal server communication.

### CX2: the standard data exchange format in the Cytoscape ecosystem

As a new web-based integration hub, Cytoscape Web requires a data exchange format that can efficiently transmit network data between Cytoscape Web and external applications. This format needs to be platform-independent, programming-language agnostic, and able to model complex data-driven visual styles and network data. Existing formats, such as Graph Modeling Language, GraphML [18], and the eXtensible Graph Markup and Modeling Language (XGMML), primarily focus on representing network topology and attributes but do not natively support data-driven visual styles or layout information. CX2 files are distinct from the Cytoscape Desktop session files that are intended to preserve the application’s full state as a Java-specific binary archive of multiple files. In contrast, CX2 is a JSON-based format jointly developed by the Cytoscape and NDEx teams to support efficient data exchange and facilitate interoperability across the Cytoscape ecosystem. In CX2, network elements and metadata are organized into separate “aspects,” each following its own schema. Typically, the set of elements stored in an aspect has the same data structure. This approach accommodates node and edge definitions, layout coordinates, and visual property mappings, allowing for dynamic, data-driven styling (e.g. node size or color) that is consistently interpreted across Cytoscape ecosystem tools. CX2’s compact design reduces memory footprint and network transfer times. Its extensibility allows new features to be incorporated without disrupting existing applications. For developers requiring custom fields, CX2 supports “opaque aspects,” enabling specialized attributes and behaviors.

To encourage widespread adoption of CX2, we integrated CX2 support into the ndex2 Python client package (https://github.com/ndexbio/ndex2-client), providing a programmatic interface for creating and managing CX2 networks. It enables efficient handling of nodes, edges, and attributes in Python and supports bidirectional conversion between CX2 networks and other data science libraries, like Pandas (https://pandas.pydata.org/) or NetworkX. Additionally, the package simplifies uploading and downloading networks to and from NDEx for persistent storage.

### System extensibility

#### App framework

Cytoscape Web extends functionality through TypeScript-based Apps while maintaining architectural alignment with Cytoscape Desktop’s plugin (App) system. Implementing desktop software plugin architecture in web applications presents unique challenges due to runtime environment and security model differences. To address these challenges, we adopted Webpack’s Module Federation framework (https://webpack.js.org/concepts/module-federation/), enabling dynamic integration of independently deployed web applications.

The framework provides developers API access to core data models—including networks, tables, visual styles, workspaces, and layout algorithms—through a provisional API that continues to evolve with community input. This implementation enables custom application development while preserving system security through a controlled distribution model that integrates trusted third-party applications via static build-time dependencies.

Through Module Federation, developers can maintain independent development and deployment cycles without synchronization with the core application’s release schedule. Developer documentation for this framework is actively maintained in the GitHub repository (https://github.com/cytoscape/cytoscape-web-app-examples).

#### Service App framework

For developers who are not comfortable with TypeScript-based modern web application development, Cytoscape Web introduces the Service App framework, which allows developers to register a REST service with Cytoscape Web to extend its functionality. The REST service can be run locally or on a remote server. At the core of the Service App framework is a REST service specification that defines endpoints for service description, task submission, required inputs, and result retrieval. The Service App REST service specification lets service developers specify the UI for inputs that their service requires without writing TypeScript code in Cytoscape Web. A Service App can be added to Cytoscape Web by simply pasting the URL in the Apps > Manage Apps … menu. Once the service is registered, Cytoscape Web will automatically process the JSON object received from the metadata endpoint of the service and generate the corresponding menu items. When a user runs the service, a dialog window appears to collect specified inputs. After the service completes, Cytoscape Web applies any actions defined by the service to the returned results. For instance, the “Update tables example” Service App (https://github.com/idekerlab/enrichment_service) applies the *updateTables* operation to the service results, which adds or updates columns in the nodes or edges table of the current network. Additional actions include opening a URL in a new tab, adding a new network, and updating a network’s selection, content, and layout. The status of installed App and Service Apps is stored in the workspace settings. It is automatically saved when the workspace is saved and restored when the workspace is reloaded.

## Results

### UI and core features

**Figure 2.**
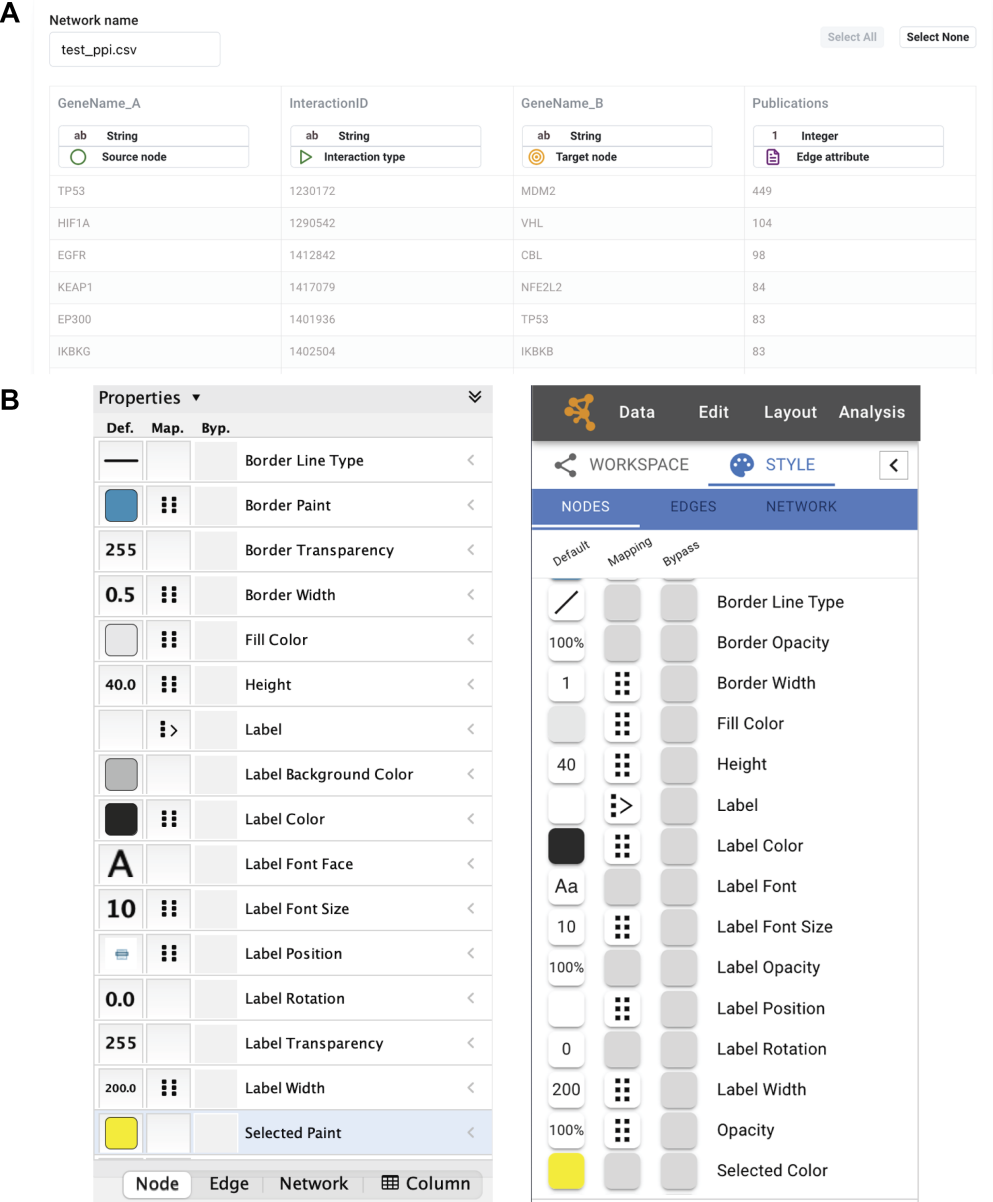
Core Cytoscape Web features. (**A**) The tabular data loader interface displays a preview of the data and allows the selection of columns to use for nodes, edges, and their attributes, including the appropriate data type. (**B**) Side-by-side comparison of the visual style editor interface in Cytoscape Web (left) and Cytoscape Desktop (right).

Cytoscape Web was designed to provide a familiar experience when performing common analysis workflows. In addition to importing existing network models from NDEx, users can create new ones starting from tabular datasets. The tabular file import interface provides a preview of the data and allows the user to define the columns to use for nodes, edges, and their attributes and, select the appropriate data type for each column (Fig. 2A). Once loaded, the data and all user-defined changes will be stored locally in the browser and will not be lost unless the Cytoscape Web’s local database is intentionally cleared by the users themselves.

The appearance of networks in Cytoscape Web can be customized using data-driven visual style mappings. The visual style editor aims to recreate its Cytoscape Desktop counterpart, allowing the creation of default, discrete, and continuous mappings as well as bypasses (Fig. 2B). Besides a minimal layout difference involving the node, edge, and network tabs position, the only substantial difference involves the visibility of the available visual style properties. In fact, while Cytoscape Desktop displays only a default selection of properties and users must manually select any additional ones they wish to use, Cytoscape Web always displays all the available properties in the Style tab, thus simplifying the end-user experience.

Once a network is laid out and styled, Cytoscape Web allows exporting raster (PNG, JPG) or vector (SVG, PDF) format images to generate high-quality figures for publications, grant proposals, or educational material.

Users can also decide to save their data to NDEx. Cytoscape Web allows users store their data in NDEx to simplify access, collaboration, and distribution of network models. While saving a network to NDEx requires users to register or log in to their accounts, this can be done directly from the Cytoscape Web interface with only a few clicks thanks to the Google Sign In feature. This feature has the advantage to persist across applications, so if users sign in when using the NDEx web app, they will also be signed in when they visit the Cytoscape Web app, and vice versa. Once the networks are saved in NDEx, users can further manage their properties to specify visibility, searchability, and provide selective access to collaborators.

In addition, Cytoscape Web enhances collaborative workflows by allowing direct sharing of networks via URLs: in fact, any network in Cytoscape Web can be shared by pasting its URL into an email or other document, like in the following example: https://web.cytoscape.org/0/networks/9a8f5326-aa6e-11ea-aaef-0ac135e8bacf?selectednodes=41536+41580+41605+41606+41719&selectededges=e42257+e42161+e42160+e42117&left=open&right=closed&bottom=open&activeTableBrowserTab=2.

The shared URL not only allows interactive access to the network in the recipient’s Cytoscape Web workspace, but also preserves some of the interface settings that the sender defined, such as a collapsed or expanded table panel, the active table panel’s tab, and the node/edge selection in the network’s graphic rendering.

Cytoscape Web currently implements core functionalities for network visualization, import/export operations, and basic filtering and visual style modifications. Certain advanced functionalities, such as detailed network analysis algorithms and a comprehensive layout library, have not yet been fully replicated. Foundational support for these features exists through a flexible App and Service App framework. This positions Cytoscape Web as an evolving platform aimed at progressively incorporating desktop capabilities over time.

### Example use case: loading and styling a network

Cytoscape Web supports loading and visualizing biological data as networks. Existing networks can be loaded from NDEx, or new ones generated by importing tabular data. Then, the appearance of the network and its layout can be modified by leveraging the data-driven styling capabilities available in Cytoscape Web. We have created a step-by-step tutorial to guide users through a common data visualization workflow. The tutorial uses the same slide deck format shared by other Cytoscape examples, and can be accessed at the following URL: https://cytoscape.org/cytoscape-tutorials/protocols/basic-data-visualization-web/index.html#/. We are also working on additional tutorials and examples that will accompany future Cytoscape Web releases.

### Example use case: visualizing the MuSIC map v1.0 with HiView

Cells exhibit multiscale organization across multiple orders of magnitude, inspiring the creation of hierarchical networks as “Cell Maps” to understand cellular structure and function. These maps contain assemblies of proteins and their interactions, arranged in nested hierarchies. It captures relationships from individual proteins and protein complexes up to pathways and organelles. However, conventional two-dimensional network visualization often fails to convey this complexity. To address this challenge, we initially developed two specialized, independent web applications, collectively called HiView [19], for visualizing Nested System in Tumors Map [20] and DNA Damage Response Assemblies Map [21]. To extend this capability to a broader community, we then implemented HiView in Cytoscape Web, an extension that enables interactive exploration and analysis of hierarchical networks.

HiView is built upon a specialized CX2-based data model called hierarchical network schema for CX2 (HCX) (https://cytoscape.org/cx/cx2/hcx-specification). HCX encodes hierarchical information, including annotations and the subnetworks that define each assembly, along with cell map specific visual styles to facilitate intuitive navigation. Using the ndex2 Python client, we transformed the original multiscale integrated cell (MuSIC) Map v1.0 [22] and its underlying protein–protein interaction data into HCX and uploaded them to NDEx. This approach enables data sharing, allowing researchers to access the MuSIC 1.0 network through a web browser without needing local installation, thereby enhancing research accessibility and reproducibility.

The MuSIC Map is among the sample networks in Cytoscape Web, and also available at this URL: https://web.cytoscape.org/0/networks/885f6d1c-96ef-11ef-af07-005056ae3c32?selectednodes=135&left=open&right=open&bottom=open&activeTableBrowserTab=0.

Once the map is loaded in Cytoscape Web, all changes made will be applied locally and not to the original network. When done working, users can save their modified copy of the map to their NDEx account. Users should not worry that their changes might be applied to the original map as the backend will not allow that to happen.

**Figure 3.**
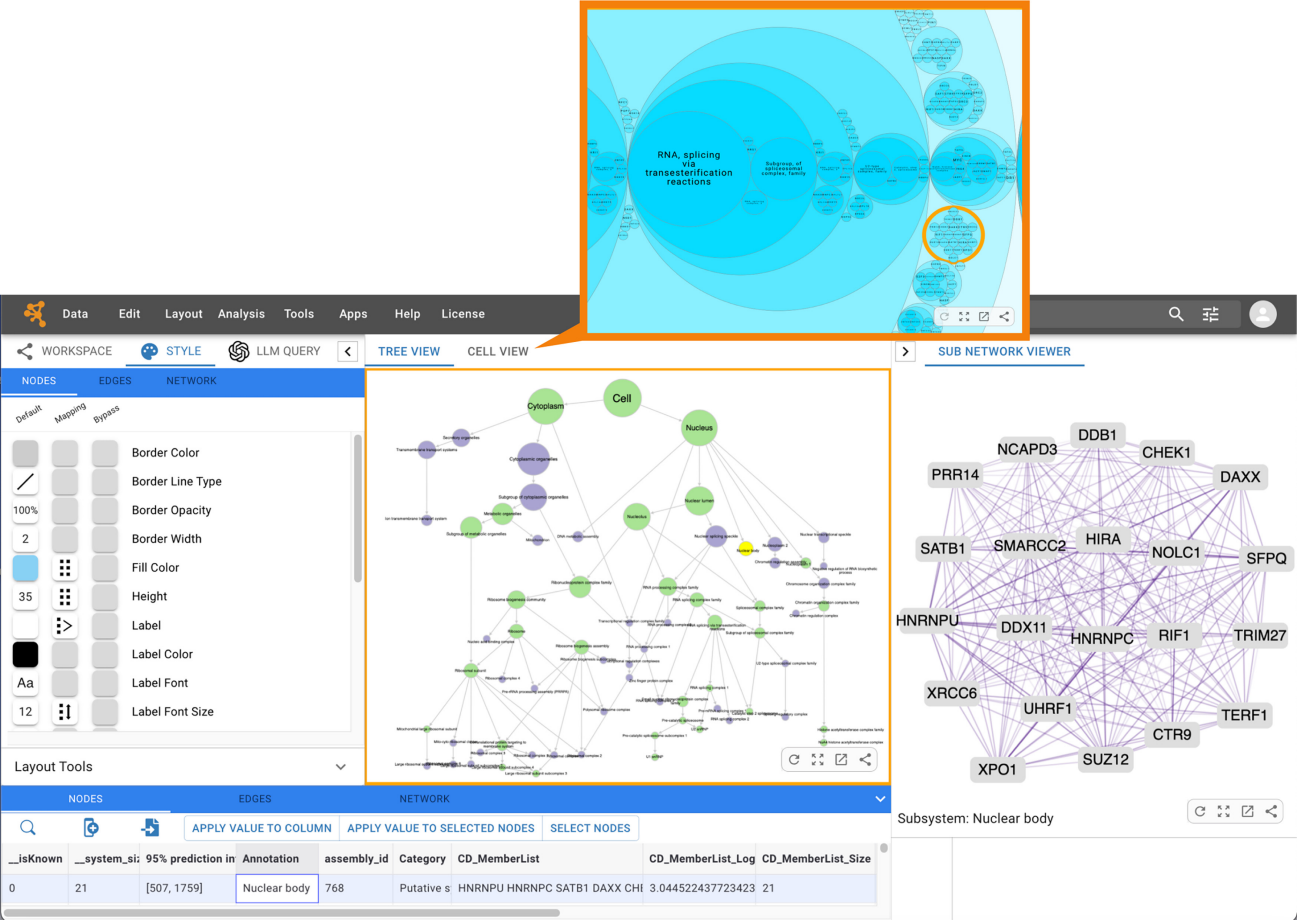
MuSIC Map v1.0 displayed in Cytoscape Web. The hierarchical map is rendered in “Tree View” by default, but users can select “Cell View” to display the model using the circle packing layout as shown in the inset. The Sub Network Viewer panel on the far right allows exploration of the underlying protein–protein interaction data for each selected subsystem.

As shown in Fig. 3, HiView introduces two new UI components that extend Cytoscape Web’s analytical capabilities. The first is the “Cell View” renderer, which employs a circle packing layout to depict hierarchical structures as nested circles. When the MuSIC Map is viewed, users can start from the top-level organelle nodes, using zoom and click to progressively increase levels of detail and gaining insights into subcellular structures.

The second component, the “Sub Network Viewer” panel, appears in a new secondary panel to the right hand side of the Cytoscape Web App interface. When a user selects an assembly in the main hierarchy, this panel displays the original supporting network, helping researchers understand the protein interactions underlying each cellular system. Other Apps can also use the same framework to integrate their own panels and extend Cytoscape Web’s functionality.

The HiView feature can be applied to any multiscale network formatted in HCX. Using the utilities available in the ndex2 Python client, researchers can easily create, modify, and upload HCX networks to NDEx. They can also define any network as a subnetwork associated with a node in the main network. Each subnetwork can include custom attributes, layouts, and styles, allowing flexible representation of complex multiscale structures. When an HCX network like the MuSIC Map is loaded, Cytoscape Web automatically activates these additional UI components, offering enhanced analysis capabilities. The MuSIC Map demonstrates how Cytoscape Web can be extended to support new data models, UIs, and visualization approaches.

### Example service app: functional enrichment

We developed two Service Apps for functional enrichment analysis that rely on g:Profiler [23, 24] and NDEx IQuery [25]. Service Apps can be added to Cytoscape Web via the Apps menu item by registering the service endpoints (the “Service App framework” section). The endpoint for g:profiler enrichment is https://cd.ndexbio.org/cy/cytocontainer/v1/gprofilerenrichment while that for IQuery enrichment is https://cd.ndexbio.org/cy/cytocontainer/v1/iqueryenrichment. The above two Service Apps require nodes in the network to have a string column/attribute in the node table, containing either a comma- or space-delimited set of gene symbols. The MuSIC v1.0 map found in the sample networks on Cytoscape Web is an example of this type of network where the nodes represent communities of genes.

The value in each network node column/attribute is therefore considered as a set of genes that serves as the basis for enrichment analysis and is processed individually using the g:Profiler or NDEx IQuery algorithms. g:Profiler analyzes gene sets against biological databases to identify statistically significant pathways, while IQuery uses the REST API provided by NDEx IQuery to retrieve pathways ranked by similarity-based matches.

**Figure 4.**
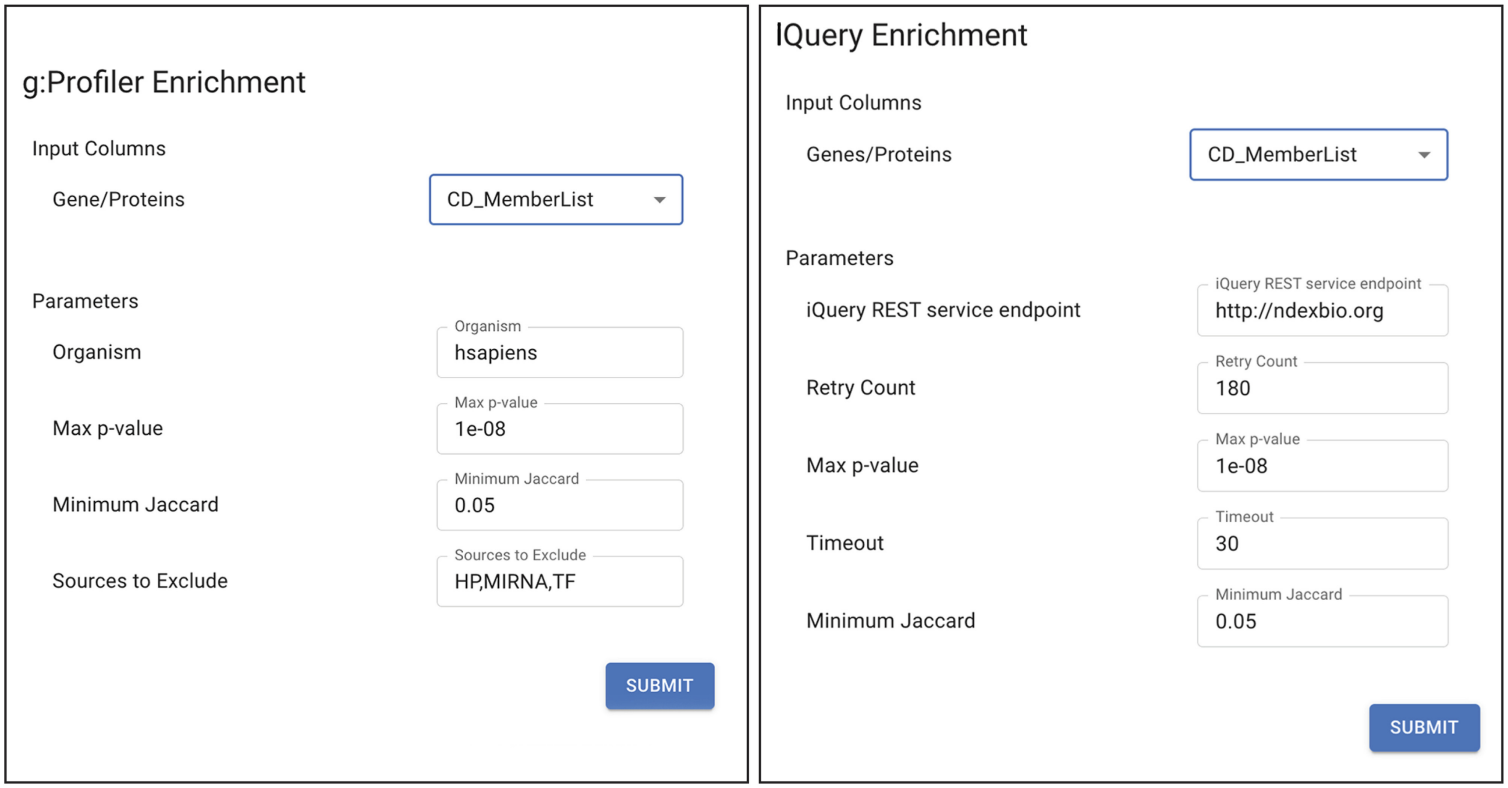
Service Apps for functional enrichment analysis. Dialog windows for the g:Profiler (left) and IQuery (right) enrichment Service Apps. The selected input column (CD_MemberList) contains the whitespace-separated set of genes used for enrichment analysis.

The dialog windows for the two Service Apps have minimal differences and are shown in Fig. 4. Once the analysis is completed, 10 new columns are added to the node table that summarize the results of the enrichment analysis. These columns include key information such as pathway name, overlap proportions, statistical significance (*P*-value), and metadata about the analysis, including the algorithm and source database used.

A detailed user manual for Cytoscape Web is available at https://web-manual.cytoscape.org. The user manual can also be conveniently accessed directly in Cytoscape Web via the “Help” menu.

## Discussion

Cytoscape Web is a browser-based framework designed to enable researchers to interact with complex datasets regardless of local hardware or operating system. This version of Cytoscape Web is a new evolution of the 2010 Flash based tool that was implemented as a tool for rendering simple networks [26]. The new Cytoscape Web is the first version to offer a significant portion of Cytoscape Desktop capabilities and is designed to support long-term development and maintenance. By following a shared URL, users can reestablish the same analysis environment—complete with data and visual configurations—on any internet-connected device. This setup encourages collaboration, as multiple team members can view and edit networks under identical conditions. Equally important, it preserves context across sessions, so researchers can seamlessly transition from one device to another while maintaining workflow continuity.

A key element of Cytoscape Web’s collaborative potential lies in its integration with the NDEx platform and the use of CX2. By storing, sharing, and publishing network models through shareable URLs, NDEx helps ensure reproducibility and transparent collaboration. Through the CX2 file format, networks retain consistent structure and styles across multiple applications, allowing users to adapt to novel data or evolving questions. This interoperability underscores the platform’s commitment to reusability and extensibility.

Cytoscape Web is designed to complement Cytoscape Desktop, offering a bidirectional pipeline for network analysis. This approach ensures that data, styles, and visual arrangements remain consistent whether researchers operate in the browser or a local desktop environment. While desktop Cytoscape excels at managing large-scale networks and offers an expansive App ecosystem, Cytoscape Web complements these strengths with immediate accessibility and built-in collaboration features. If a new App appear in one setting only, networks can still be transferred between the two platforms, allowing researchers to harness emerging methods, even if they are exclusive to one environment. We anticipate that many users will prefer the web platform for simpler use cases, and we remain committed to supporting and evolving both the web and desktop applications in the future.

Cytoscape Web offers a framework for integrating with existing workflows, web applications, and desktop pipelines. Its App and Service App architectures, combined with a URL-based import feature, allow external tools to hand off networks directly to Cytoscape Web as long as they can serve CX2-formatted data via a REST endpoint. For example, if a Jupyter notebook produces a CX2 file at http://localhost:8080/xyz/mytempresult.cx2, simply navigating to https://web.cytoscape.org?import=http://localhost:8080/xyz/mytempresult.cx2 automatically loads the resulting network in Cytoscape Web. This streamlined approach ensures that advanced features—such as hierarchical “Cell View” visualizations, broad style and table editing, and cloud-based collaboration—are accessible within diverse research environments.

### Limitations and future plans

A known limitation of Cytoscape Web is related to memory allocation. Most browsers allocate between 1 and 2 GB of memory per tab, depending on the browser and system. Cytoscape Web must operate within these constraints, which can limit its ability to handle large networks. As a temporary workaround, Cytoscape Web can only open networks that contain up to 20 000 edges or 26 000 edges and nodes combined, whose cx2 file size is <500 MB, which covers the majority of biological networks at present (for larger networks users are referred to Cytoscape Desktop). Performance issues are being actively addressed through ongoing efforts to integrate advanced technologies like WebGL (https://www.khronos.org/webgl/) in Cytoscape.js and custom rendering components using Deck.gl (https://deck.gl/).

While Cytoscape Web provides the essential functionality of Cytoscape Desktop, many advanced features, such as undo capabilities, image annotations for styles, the Network Analyzer, programmatic access with languages like Python and R, and additional data import options (e.g. .sif, .xlsx), are currently missing. Plans are in place to implement these features to bring Cytoscape Web closer to feature parity with its desktop counterpart. Furthermore, browser dependency introduces constraints; the tool’s performance varies with the capabilities of different browsers, and its reliance on an active internet connection makes offline use infeasible. To mitigate these limitations, the development team is exploring ways to optimize browser-based workflows and enhance the user experience. Additionally, the team recognizes the future potential of making Cytoscape Web embeddable as a view-only widget for integration into external websites, leveraging its collaborative advantages for network sharing. Maintenance plans also include iterative improvements to the UI, scalability of cloud services like NDEx, and developer-facing APIs to ensure a robust experience for all users.

Access to external interaction databases is a key feature of the Cytoscape desktop application and relies on apps that have not been ported to Cytoscape Web yet. Plans are in place to add this functionality in future releases. NDEx hosts several interaction databases such as STRING, BioGRID, HumanNet, and more. Users can find and query these large interactomes directly in NDEx and save the results of their queries. Once the query results are saved to the user's NDEx account, they can be opened in Cytoscape Web for further manipulation.

## Supplementary Material

gkaf365_Supplemental_File

## Data Availability

Cytoscape Web is publicly accessible at https://web.cytoscape.org. It is free and open to all users. Upload and analysis of sensitive personal information requires a login. The source code, distributed under the MIT license, is available in the following repositories: • Cytoscape Web: https://github.com/cytoscape/cytoscape-web • All releases are archived in Zenodo under the DOI https://doi.org/10.5281/zenodo.14775458 • Example Apps: https://github.com/cytoscape/cytoscape-web-app-examples
