# Supplementary material for: Cytoscape Web: bringing network biology to the browser
Source: Nucleic Acids Res. 2025 May 1;53(W1):W203–12. doi: 10.1093/nar/gkaf365 (PMC12230733; doi:10.1093/nar/gkaf365)
Supplement: gkaf365_Supplemental_File [file gkaf365_supplemental_file.pdf]

SUPPLEMENTARY DATA

Supplementary Data are available at NAR online.

Technology Stack

Cytoscape Web leverages modern web technologies to deliver interactive network visualization and analysis capabilities. TypeScript (https://www.typescriptlang.org/) serves as the primary programming language, ensuring type safety and minimizing runtime errors in team-based collaborative development. The build process uses Webpack (https://webpack.js.org/), implementing Module Federation to enable extensible and distributed application architecture.

The user interface is built on React (https://react.dev/), with Zustand (https://github.com/pmndrs/zustand) managing global state and Material UI (https://mui.com/material-ui/) providing consistent interface components. Network visualization capabilities integrate multiple specialized libraries: Cytoscape.js (https://js.cytoscape.org/) serves as the primary renderer, while D3.js (https://d3js.org/) enables hierarchical data visualization through Circle Packing. High-performance graph layouts are achieved through GPU-accelerated engines including Cosmos (https://github.com/cosmograph-org/cosmos) and G6 (https://g6.antv.antgroup.com/en/). Local data persistence is implemented via IndexedDB with Dexie.js (https://dexie.org/), enabling robust workspace caching and ensuring data reliability without continuous server communication.

This technology stack provides a robust foundation for web-based network visualization and data exchange, while ensuring system extensibility and performance optimization.

Documentation

This section lists all the technical documentation relevant to Cytoscape Web.

- Cytoscape Web developer's guide: https://github.com/cytoscape/cytoscape-web/wiki/Tutorial-For-Cytoscape-Web
- CX2 specification: https://cytoscape.org/cx/cx2/specification/cytoscape-exchange-format-specification-(version-2)/
- CX2 visual styles: https://cytoscape.org/cx/cx2/cx2-visual-styles/
- Hierarchical network schema for CX2: https://cytoscape.org/cx/cx2/hcx-specification/
- Service App Specification: https://github.com/cytoscape/cytoscape-web/wiki/Specification-for-Service-App-in-Cytoscape-Web-(draft-v2)
- User manual: https://web-manual.cytoscape.org

- Cytoscape Web Development Priorities: https://github.com/cytoscape/cytoscape-web/wiki/Cytoscape-Web-Development-Priorities
- Cytoscape Web vs Desktop Feature: Comparison:https://github.com/cytoscape/cytoscape-web/wiki/Cytoscape-Web-vs-Desktop-Feature-Comparison
